# FAM20A binds to and regulates FAM20C localization

**DOI:** 10.1038/srep27784

**Published:** 2016-06-13

**Authors:** Yoshio Ohyama, Ju-Hsien Lin, Nattanan Govitvattana, I-Ping Lin, Sundharamani Venkitapathi, Ahmed Alamoudi, Dina Husein, Chunying An, Hak Hotta, Masaru Kaku, Yoshiyuki Mochida

**Affiliations:** 1 Department of Molecular and Cell Biology, Henry M. Goldman School of Dental Medicine, Boston University, Boston, United States of America; 2 Research Unit of Mineralized Tissue, Faculty of Dentistry, Chulalongkorn University, Bangkok, Thailand; 3 Division of Microbiology, Kobe University Graduate School of Medicine, Kobe, Japan; 4 Division of Bio-Prosthodontics, Niigata University Graduate School of Medical and Dental Sciences, Niigata, Japan

## Abstract

Mutations in the *Family with sequence similarity (FAM) 20* gene family are associated with mineralized tissue phenotypes in humans. Among these genes, *FAM20A* mutations are associated with Amelogenesis Imperfecta (AI) with gingival hyperplasia and nephrocalcinosis, while *FAM20C* mutations cause Raine syndrome, exhibiting bone and craniofacial/dental abnormalities. Although it has been demonstrated that Raine syndrome associated-*FAM20C* mutants prevented FAM20C kinase activity and secretion, overexpression of the catalytically inactive D478A FAM20C mutant was detected in both cell extracts and the media. This suggests that FAM20C secretion doesn’t require its kinase activity, and that another molecule(s) may control the secretion. In this study, we found that extracellular FAM20C localization was increased when wild-type (WT), but not AI-forms of FAM20A was co-transfected. On the other hand, extracellular FAM20C was absent in the conditioned media of mouse embryonic fibroblasts (MEFs) derived from *Fam20a* knock-out (KO) mouse, while it was detected in the media from WT MEFs. We also showed that cells with the conditioned media of *Fam20a* WT MEFs mineralized, but those with the conditioned media of KO MEFs failed to mineralize *in vitro*. Our data thus demonstrate that FAM20A controls FAM20C localization that may assist in the extracellular function of FAM20C in mineralized tissues.

FAM20A, FAM20B and FAM20C are the three structurally-related members of the “family with sequence similarity 20” (FAM20) proteins^1^. Non-sense or missense mutations in the *FAM20A* gene were identified that are associated with an autosomal recessive type of Amelogenesis Imperfecta (AI), resulting in premature termination or amino acid changes of the peptides (OMIM#614253). These AI patients with *FAM20A* mutations have several major dental abnormalities such as generalized hypoplastic enamel, intrapulpal calcification, delayed tooth eruption, and failure of tooth development as well as gingival hyperplasia^2^,^3^. More recently, a non-dental phenotype of nephrocalcinosis has also been reported in the AI patients with *FAM20A* mutation, further indicating the potential molecular function of FAM20A in biomineralization^4^,^5^,^6^,^7^,^8^,^9^. FAM20B encodes a protein kinase that phosphorylates xylose residue in the tetrasaccharide glycosaminoglycan (GAG)-protein linkage region in the Golgi apparatus^10^, thereby regulating the subsequent GAG chain assembly^11^. Proteoglycans derived from *FAM20B* TALEN knock-out (KO) cells were chondroitin and heparin sulfate GAGs-deficient^11^, which likely explains why the *Fam20b* deficient zebrafish exhibited poor cartilage matrix production *in vivo*^12^. *FAM20C* was found to be a causative gene for Raine syndrome (OMIM#259775) that manifests pulmonary hypoplasia, osteosclerosis, craniofacial dysmorphism, dental anomalies and gingival hyperplasia^13^,^14^,^15^. The mice deficient for *Fam20c* exhibit a systemic hypophosphatemic condition likely by increasing serum levels of the crucial phosphorus regulating hormone FGF23^16^. Dental phenotypes in these mice have also been reported^17^,^18^,^19^. Recent reports demonstrated the multi-functionality of FAM20C located in the Golgi and extracellular space. It has been reported that FAM20C acts as the Golgi Casein Kinase (GCK) that phosphorylates casein and the Small Integrin-Binding Ligand, N-linked Glycoproteins (SIBLINGs) which are critical for biomineralization^20^,^21^. Addition of recombinant FAM20C protein into cell cultures promotes osteoblast differentiation^16^, indicating the potential role of extracellular FAM20C as a growth and differentiation factor-like protein.

Currently, there is a debate as to whether endogenous FAM20C is secreted or secretion is an artifact of overexpression in cell culture system^22^,^23^, thus it is important to determine whether endogenous FAM20C is present in the extracellular space and also to delineate the mechanism how FAM20C secretion is controlled. It has been demonstrated that secretion of FAM20C (by overexpression) occurs regardless of its kinase activity, because a catalytically inactive FAM20C (D478A) mutant, but not most of other Raine syndrome-associated missense FAM20C mutants, is still detected in the conditioned media^23^. This suggests the presence of another molecule(s) which likely determines the FAM20C localization. Therefore, the purpose of our current study was to identify and investigate the molecule(s) that can regulate FAM20C secretion. Here we show that FAM20A binds to FAM20C in cell cultures and *in vitro*. When FAM20C is transiently overexpressed together with wild-type (WT) FAM20A in 293 cells, FAM20C secretion is increased as compared to cells in which FAM20C is expressed alone. FAM20A forms mimicking AI-mutations fail to enhance the FAM20C secretion. Similarly, extracellular FAM20C is absent in mouse embryonic fibroblast (MEF) cultures derived from *Fam20a* KO mice, but present in cultures derived from WT MEFs, demonstrating that FAM20A is required for FAM20C extracellular localization. To assess the biological importance of FAM20A-controled FAM20C secretion, *in vitro* mineralization assay was performed using MC3T3-E1 osteoblastic cells treated with the conditioned media derived from *Fam20a* KO or WT MEF cells. Our results showed that the extent of mineralization of MC3T3-E1 cells treated with the conditioned media from *Fam20a* KO MEF cells was markedly lesser than that of MC3T3-E1 cells with the conditioned media from *Fam20a* WT MEF cells. To the best of our knowledge, our data demonstrate for the first time that FAM20A regulates FAM20C localization and assists in the extracellular function of FAM20C.

## Results

### Expression of FAM20 family members in various tissues

**Figure 1:**
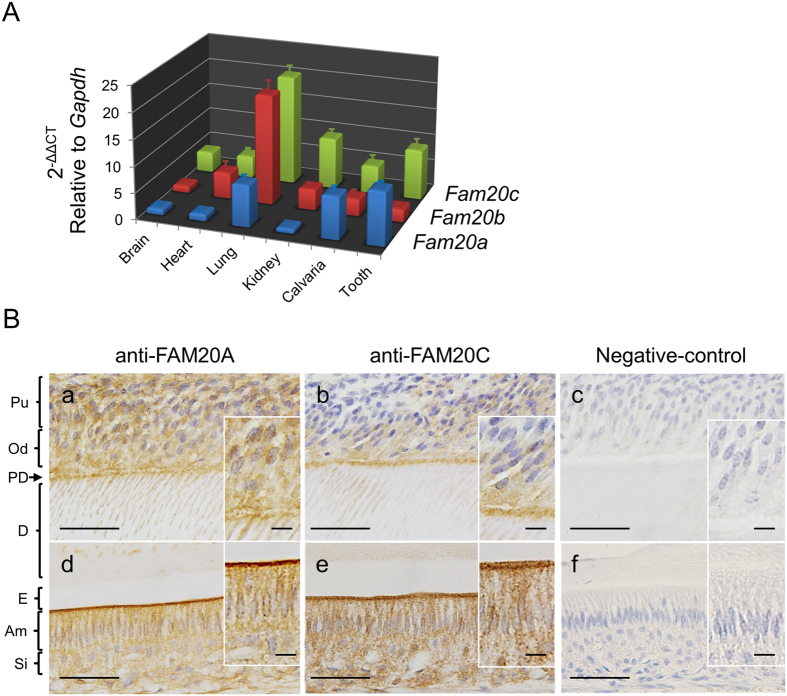
Expression of *Fam20* family members in various tissues and presence of FAM20A and FAM20C proteins in odontogenic cells. (**A**) Comparison of gene expression among *Fam20* family members. Quantitative real-time PCR analysis was performed using 5-week-old mouse tissues (brain, heart, lung, kidney, calvaria, tooth). The mean fold change in the expression of each *Fam20* member was calculated based on the normalization to that of *glyceraldehyde-3-phosphate dehydrogenase* (*Gapdh*) using the value of *Fam20a* in brain as a calibrator. The values are shown as the mean + S.D. based on triplicate assays. The expression of *Fam20a* is indicated by blue bars, *Fam20b* by red and *Fam20c* by green. (**B**) Immunohistochemical analysis of FAM20A and FAM20C in adult mouse incisors. FAM20A (a,d) and FAM20C (b,e) were both localized in the same cell/tissue types. No immunoreactivities were observed when non-immune goat serum was used as a negative control (c,f). Scale bar, 50 μm. Images of odontoblasts and ameloblasts were shown at a higher magnification on the right corner of each image (scale bar, 10 μm). Pu; pulp, Od; odontoblasts, PD; pre-dentin, D; dentin, E; enamel, Am; ameloblasts, Si; stratum intermedium.

To investigate the gene expression of all FAM20 family members in various mouse tissues, real-time PCR was performed (Fig. 1A, numerical data in S1 Table). The results demonstrated that the patterns of gene expression in heart, lung and kidney were similar among *Fam20* genes, i.e. the expression of *Fam20b* (red) and *Fam20c* (green) was higher than that of *Fam20a* (blue), while the patterns of gene expression in calvaria and tooth were similar, i.e. the expression of *Fam20a* and *Fam20c* was higher than that of *Fam20b*.

### Localization of FAM20A and FAM20C proteins in odontogenic cells

Since mutations in *FAM20A* as well as *FAM20C* manifest dental phenotypes, our results led to investigate whether FAM20A and FAM20C proteins are expressed in odontogenic tissues. Immunohistochemical analysis of adult mouse incisors using anti-FAM20A and anti-FAM20C antibodies was performed (Fig. 1B). The results demonstrated that both FAM20A (left panels) and FAM20C (middle panels) proteins were localized in odontogenic cells/tissues including pulp, odontoblasts, ameloblasts and stratum intermedium with granular patterns of the immunoreactivity. Both FAM20A and FAM20C proteins appeared to be accumulated at pre-dentin, dentinal tubules within odontoblasts and Tome’s processes of ameloblasts. No immunoreactivity was observed when non-immune goat serum was used (right panels). Similar localization of FAM20A to FAM20C led us to investigate the potential interaction between these proteins.

### FAM20A binds to FAM20C

**Figure 2:**
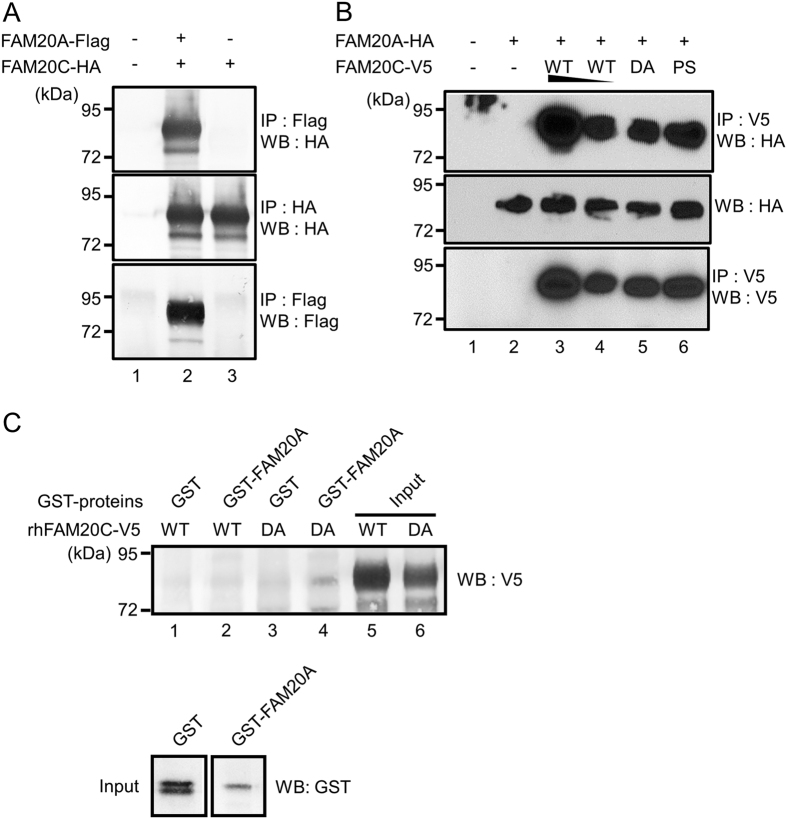
FAM20A directly binds to FAM20C. (**A**) Binding assay in a cell culture system. The 293 cells were transiently transfected with FAM20A-Flag (lane 2) and FAM20C-HA (lanes 2 and 3). Cell lysates were prepared and immunoprecipitated (IP) with anti-Flag antibody. The interaction was detected by Western blotting (WB) with anti-HA antibody (upper panel). Presence of FAM20C-HA (middle panel) and FAM20A-Flag (lower panel) in the same lysates was verified. (**B**) FAM20A-FAM20C interaction is independent from FAM20C kinase activity. The 293 cells were transfected with FAM20A WT-HA and FAM20C WT-V5 (WT), FAM20C D478A-V5 (DA), or FAM20C P328S-V5 (PS). Cell lysates were immunoprecipitated with anti-V5 antibody and the interaction was detected by WB analysis with anti-HA antibody (upper panel). Presence of FAM20A WT-HA (middle panel) and FAM20C forms (lower panel) in the same lysates was confirmed. (**C**) Binding assay by GST pull down. The rhFAM20C WT-V5/His or rhFAM20C D478A-V5/His protein was incubated with either GST protein alone or GST-FAM20A protein and coupled to glutathione beads. After washing the beads, the bound proteins were visualized by WB analysis with anti-V5 antibody (upper panel). Presence of rhFAM20C WT-V5/His (upper panel, lane 5), rhFAM20C D478A-V5/His (upper panel, lane 6), GST and GST-FAM20A (lower panels) proteins were assessed by WB analysis.

Since the localization of FAM20A was similar to that of FAM20C in tooth, and clinical phenotypes of AI patients with *FAM20A* mutation partially overlap with those of Raine syndrome patients, we investigated the association of FAM20A with FAM20C. The FAM20C expression vector with C-terminal HA tag (pc3-FAM20C WT-HA) was transfected together with pc3-FAM20A WT-Flag into 293 cells, and the interaction was assessed. The results clearly showed that FAM20A interacted with FAM20C (Fig. 2A, upper panel, lane 2). Furthermore, two types of FAM20C mutant forms (D478A and P328S) were tested to determine that this interaction requires FAM20C kinase activity. As shown in Fig. 2B, the interaction between FAM20A and FAM20C was again confirmed in a dose dependent manner with a different combination of the tag used from Fig. 2A (Fig. 2B, upper panel, lanes 3 and 4). We also found that two of FAM20C mutant forms still bound to FAM20A, demonstrating that FAM20A-FAM20C interaction occurs in a FAM20C-kinase activity independent manner (Fig. 2B, upper panel, lanes 5 and 6).

To determine whether or not the binding observed was direct (i.e. without the presence of other molecules), we initially examined the *in vitro* binding by incubating rhFAM20C WT-V5/His protein purified from conditioned media of stably transfected 293 cells with recombinant GST-FAM20A fusion protein produced in *E. coli* and purified, followed by a GST pull down assay. However, the binding between FAM20A and FAM20C was not clearly observed. We reasoned that a protein kinase didn’t usually associate firmly with substrate-like proteins^24^. It has been previously reported that the mutation of the aspartate residue at 478 in FAM20C abolished its intracellular kinase activity^20^, consistent with the requirement of a divalent cation binding to this residue for the catalysis^25^. We thus investigated the *in vitro* binding assay using rhFAM20C D478A-V5/His protein and tested this possibility (Fig. 2C, upper panel, lanes 1–4). The expression of rhFAM20C WT-V5/His (Fig. 2C, upper panel, lane 5), rhFAM20C D478A-V5/His (Fig. 2C, upper panel, lane 6), GST and GST-FAM20A in extracts used for the pull down (Fig. 2C, lower panels) are shown in Western blots of the input samples. GST alone did not bind to rhFAM20C WT-V5/His and rhFAM20C D478A-V5/His (Fig. 2C, upper panel, lanes 1 and 3). When incubated with GST-FAM20A, rhFAM20C D478A-V5/His (Fig. 2C, upper panel, lane 4), but not rhFAM20C WT-V5/His (Fig. 2C, upper panel, lane 2) showed the binding. These results suggest that FAM20C is a direct binding partner of FAM20A.

### FAM20A regulates FAM20C localization

**Figure 3:**
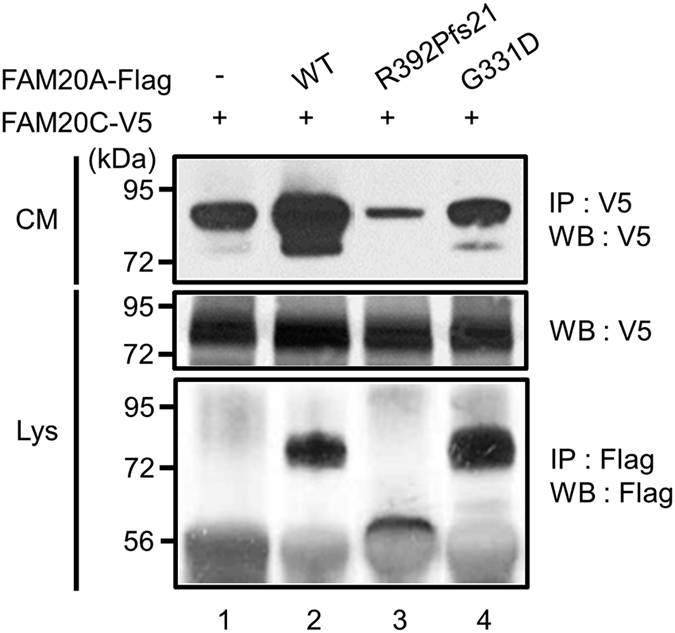
Wild-type, but not AI-mutated forms of FAM20A, is necessary for extracellular FAM20C accumulation. The 293 cells were transfected with pc3-FAM20A WT-Flag (WT), pc3-FAM20A R392Pfs21-Flag (R392Pfs21) or pc3-FAM20A G331D-Flag (G331D) together with pc3.1-FAM20C WT-V5/His (FAM20C-V5). Conditioned media (CM) were collected and IP-WB analysis was performed to detect extracellular FAM20C (upper panel). The intracellular expression of FAM20C was verified by WB analysis with cell lysates (Lys, middle panel) and that of FAM20A WT and AI-mutants was also confirmed by IP-WB analysis with cell lysates (lower panel). FAM20C secretion is accelerated with FAM20A WT co-transfection as compared to FAM20C transfection alone (upper panel, lane 2 vs. 1ane 1). AI-associated FAM20A mutants, R392Pfs21 (upper panel, lane 3) and G331D (upper panel, lane 4), fail to enhance the extracellular FAM20C accumulation.

During the course of our investigation on the effect of FAM20A on FAM20C expressed in 293 cells, we found that the amount of secreted FAM20C was greatly enhanced in the presence of FAM20A-WT as compared to FAM20C expressed alone (Fig. 3, upper panel, lane 2 vs. lane 1). We next investigated whether the mutated forms of FAM20A associated with AI similarly enhance the amount of secreted FAM20C. As shown in Fig. 3, the level of extracellular FAM20C was not enhanced or even inhibited when AI-associated FAM20A mutant, R392Pfs21 or G331D was co-transfected (upper panel, lane 3 or lane 4, respectively) as compared to FAM20C alone (upper panel, lane 1). The expression levels of intracellular FAM20C were unchanged (middle panel, lanes 1–4). The results indicate that wild type FAM20A is necessary for FAM20C extracellular accumulation and that AI-associated FAM20A forms appear to fail to enhance FAM20C secretion.

**Figure 4:**
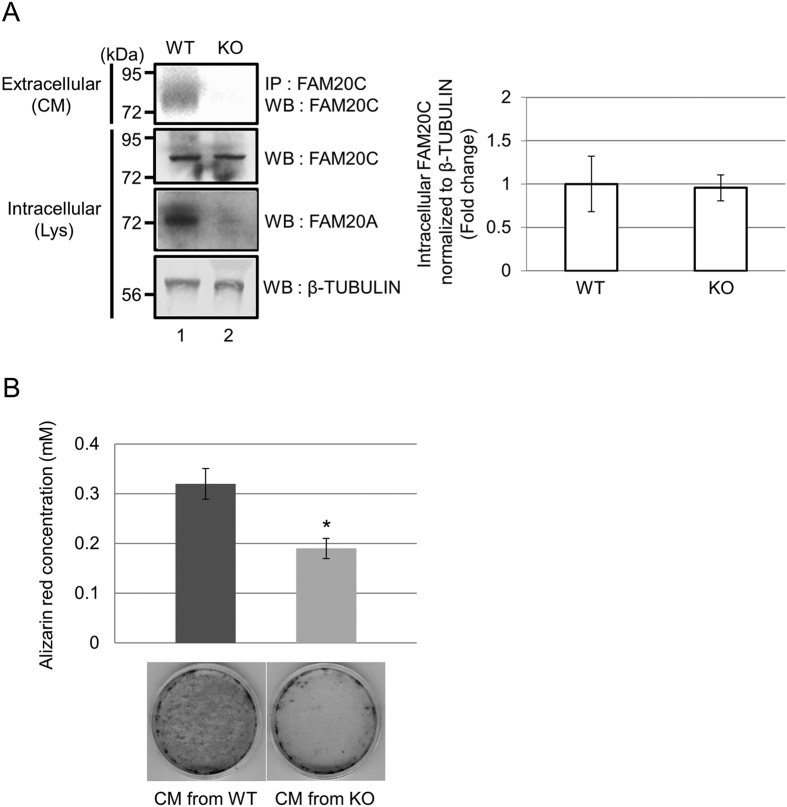
FAM20A is required for extracellular FAM20C localization and impact of FAM20A-mediated FAM20C secretion on *in vitro* mineralization in osteoblasts. (**A**) Conditioned media and cell lysates were collected from Wild-type (WT) and knockout (KO) of *Fam20a*-KO mouse-derived MEF cells. IP-WB analysis with anti-FAM20C antibody was performed to detect extracellular FAM20C (top panel). Intracellular FAM20C (upper middle panel) and intracellular FAM20A (lower middle panel) were detected by WB analysis with anti-FAM20C and anti-FAM20A antibodies, respectively. The expression of β-TUBULIN was shown (bottom panel) as a loading control. Extracellular FAM20C was not detected in KO MEF cells, although intracellular FAM20C levels were comparable between WT and KO cell cultures. Three independent experiments were performed and the representative set of images were shown. The band intensities of intracellular FAM20C normalized to β-TUBULIN in the same lysate were calculated and the values are expressed as the mean + SD (n = 3) (graph on the right). There was no statistical difference of normalized intracellular FAM20C expression between WT and KO. (**B**) MC3T3-E1 osteoblastic cells were cultured with conditioned media (CM) from *Fam20a* WT and KO MEF cells, and impact of FAM20A-mediated FAM20C secretion was assessed by *in vitro* mineralization assay. Mineralized nodule formation was impaired in osteoblasts the presence of CM from *Fam20a* KO MEF cells. Three independent experiments were performed and the representative set of images were shown. The concentration of Alizarin Red S dye extracted from each cell culture matrix was calculated and the values are expressed as the mean + SD (n = 3) with statistical analysis. The asterisk above the bar graph indicates the presence of statistical difference between CM from WT and CM from KO treatment groups. *p < 0.01.

In order to determine whether the data obtained in the 293 cell model reflects what occurs in primary cells, we further examined whether or not extracellular FAM20C is present in cultures of *Fam20a* KO MEF cells. Conditioned media and cell lysates of MEF cells derived from WT and KO embryos were collected and the levels of extracellular FAM20C, and intracellular FAM20C and FAM20A were evaluated using anti-FAM20C and anti-FAM20A antibodies. Our results demonstrated that extracellular FAM20C was found in the conditioned media from WT MEF cells (Fig. 4, upper panel, lane 1), however, FAM20C was absent in KO (Fig. 4, upper panel, lane 2). The expression level of intracellular FAM20C was normalized to that of β-TUBULIN and the normalized value of intracellular FAM20C in the cell lysate from KO was similar to that of WT (Fig. 4A, graph on the right). The expression of intracellular FAM20A was clearly observed in WT (Fig. 4, lower middle panel, lane 1), but not in KO (Fig. 4, lower middle panel, lane 2), confirming the absence of FAM20A protein in *Fam20a* KO-derived cells. The results clearly show that FAM20A is required for FAM20C secretion.

To further determine the biological importance of FAM20A-controlled FAM20C secretion, the effects of conditioned media from *Fam20a* WT and KO MEF cells on *in vitro* mineralization in MC3T3-E1 osteoblastic cells were investigated. MC3T3-E1 cells were plated, cultured with either conditioned media (CM) from *Fam20a* WT or KO MEF cells, and mineralized nodules were stained with Alizarin Red S dye. The results showed that MC3T3-E1 cells cultured with CM from *Fam20a* KO MEF cells failed to mineralize, while cells cultured with CM from *Fam20a* WT fully mineralized. The Alizarin Red S dye was extracted from the mineralized nodules deposited in cell cultures and the concentration of the dye was quantified. The values extracted from MC3T3-E1 cells with CM from *Fam20a* WT were significantly greater than those with CM from *Fam20a* KO (Fig. 4B), indicating that *Fam20a* is required for FAM20C-mediated mineralized nodule formation in osteoblasts.

## Discussion

*FAM20A* was originally identified as an upregulated gene in murine bone marrow cells with a retrovirus expressing a dominant negative retinoic acid receptor α (RARα) protein, and public database searches also identified *FAM20B* and *FAM20C* by similarity^1^. *FAM20B* is evolutionally different from the other 2 members, as this gene has been conserved from *Caenorhabditis elegans* to human, while *FAM20A* and *FAM20C* have been identified from *Fugu rubripes* (pufferfish) to human^1^. Gene knockout study also suggests that the phenotype of *Fam20b* is distinct and different from the other 2 members; *Fam20b* KO mice show embryonic lethality at E12.5, while *Fam20a* and *Fam20c* KO mice do not^26^. Our data in Fig. 1 demonstrate that the expression patterns of *Fam20a* and *Fam20c* are similar and well correlated with the tissues where clinical phenotypes in AI with *FAM20A* mutation and Raine syndrome were found, while that of *Fam20b* is different. The evolutionary conservation, gene KO phenotype and differential expression among FAM20 members may suggest that the molecular functionality of FAM20A is associated with FAM20C, but not FAM20B. This notion is also confirmed by the recent report that FAM20A forms a protein complex with FAM20C and regulates FAM20C kinase activity^27^.

There are currently conflicting results reported whether FAM20A, FAM20B and FAM20C are secretory proteins or intracellular Golgi-proteins or both^1^,^20^,^21^,^28^,^29^. Overexpression of FAM20 proteins have been previously performed in several studies. The expression of FAM20A^1^,^20^ and FAM20B^20^ was identified in both cell lysate and conditioned media, while it was detected only in the cell lysate in another report^21^. FAM20C secretion by overexpression or at the endogenous level was observed in all studies tested^1^,^20^,^21^,^27^,^30^. Although less quantitative, endogenous FAM20 protein immunostaining has been shown in skeletal/dental tissues. The immuno-localization of FAM20A^2^,^6^ and FAM20B^2^ were observed only within cells, while that of FAM20C detected in both extracellular matrices and cells^2^,^28^. Our immunohistochemical data demonstrated that the immunoreactivities to anti-FAM20C antibody were mainly observed intracellularly, in the cytoplasmic areas of odontogenic cells including odontoblasts, ameloblasts, stratum intermedium and pulp with granular patterns. Our immunohistochemical analysis also demonstrated the presence of FAM20C in pre-dentin, which may suggest that secretion of FAM20C occurs in a tissue-specific manner.

It is not clear, at this point, why FAM20A directly binds to a kinase-inactive form, but not WT of FAM20C (Fig. 2B) although FAM20A binds to FAM20C WT in cell cultures (Fig. 2A,B). It is generally accepted that the kinase doesn’t always stably associate with its corresponding substrate because the biochemical phosphorylation reaction is transient^31^ and this transient contact typically prevents or at least strongly reduces the efficacy of the substrate detection by the commonly used protein-protein interaction methods^24^, suggesting the elaboration of biochemical techniques is required to identify kinase targets. This concept may be the basis for the direct binding between wild type FAM20A and FAM20C was not detected, while FAM20C D478A (inactive kinase) did co-immunopreciptate with FAM20A. Very recently, it has been demonstrated that FAM20A functions as a pseudokinase in a complex with FAM20C^27^. Molecular function of some pseudokinases has been studied and reported^32^. STE20-related pseudokinase, STRADα, for example, forms a protein complex with a protein kinase LKB1^33^, resulting in the activation of LKB1 kinase activity. The kinase activity and localization of LKB1 was further enhanced with MO25, the scaffold molecule with STRADα^34^. We showed that the mutated forms of FAM20A failed to enhance extracellular FAM20C accumulation, which is consistent with the recent report that FAM20A binds to ATP, which may stabilize FAM20A conformation^27^.

Although the biochemical function of FAM20A in conjunction with FAM20C GCK activity was demonstrated, questions especially *in vivo* phenotypes still remain. *Fam20c* knockout mice exhibit hypophosphatemia due to the inability to properly control FGF23 secretion, however the levels of Ca and phosphate in *Fam20a* knockout mice were normal^26^. This raises a possibility that FAM20A may function in FAM20C-independent manner. Consistent with this notion, there are broad and distinct clinical features in AI patients with *FAM20A* mutation (OMIM#614253) and *Fam20a* KO models^26^,^35^. Previous studies suggested that the mineralization inhibitor(s) may be FAM20A’s potential substrate(s), because *FAM20A* mutations in human or *Fam20a* deficiency in mouse KO models are associated with ectopic calcification phenotypes (Loss of FAM20A kinase activity, if any, may result in loss of phosphorylation in these molecules and thus loss of inhibition)^4^,^5^,^6^,^26^,^36^,^37^. Among these inhibitors, it has been reported that alpha-2-HS-glycoprotein (also known as fetuin) deficiency resulted in the ectopic calcification phenotype^38^, resembling to *Fam20a* KO mice^26^. However, the finding that FAM20A is a pseudokinase conflicts with this idea. Thus, it is an interesting future study to delineate molecular mechanism(s) by which the ectopic calcification phenotype occurs in *Fam20a* KO mice.

**Figure 5:**
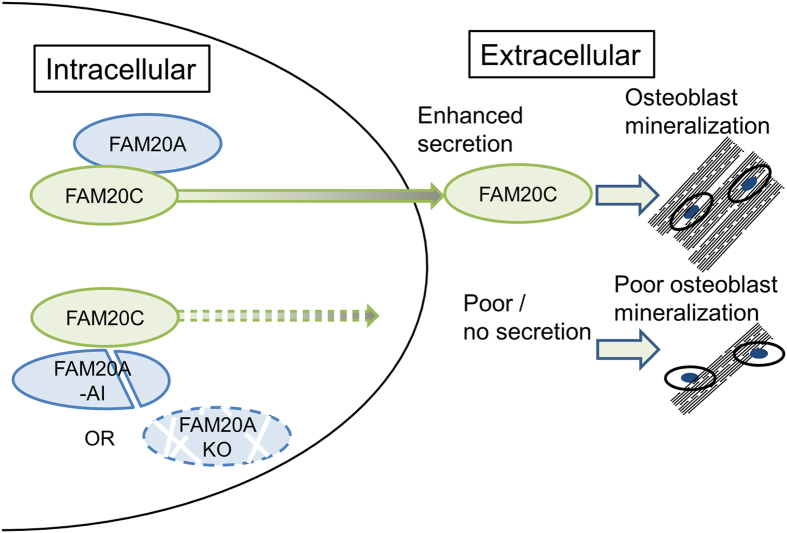
Schematic representation of FAM20A-FAM20C interaction and FAM20A-mediated FAM20C secretion. A model for the role of FAM20A in the FAM20A-FAM20C complex. FAM20A binds to FAM20C, in which FAM20C may be allosterically modulated by FAM20A, leading to secretion. In the case of FAM20A with Amelogenesis Imperfecta (AI) mutation or absence of FAM20A (i.e. KO), FAM20C secretion does not occur, which consequently results in poor osteoblast mineralization.

In the present study, we show, for the first time, that FAM20A regulates FAM20C secretion into the extracellular space and propose a model in Fig. 5. It is of particular interest that DMP4, a mouse homolog of FAM20C was originally identified as an extracellular calcium-binding protein which promotes HAT-7 ameloblastic cell mineralization^30^. Addition of FAM20C into MC3T3-E1 osteoblast cell cultures up-regulates the expression of several bone differentiation markers such as *Bone sialoprotein* (*BSP*), *Dentin matrix protein* (*DMP*)*1* and *Osteocalcin*^16^, indicating that extracellular FAM20C functions as a growth and differentiation factor in mineralizing cells. Therefore, the regulation of FAM20C secretion by FAM20A is crucial for mineralized tissue biology. In support with this notion, our results showed that addition of the conditioned media from *Fam20a* KO MEF cell cultures (which had under detection levels of FAM20C) failed to form mineralized nodules in MC3T3-E1 osteoblastic cultures *in vitro* (Fig. 4B).

As the emerging FAM20 kinase family functions become clear, the effect of FAM20A on FAM20C localization may define the novel mechanisms of mineralized tissue biology at a molecular level and is currently under investigation in our laboratory.

## Materials and Methods

### Ethics statement

The use of animals and all animal procedures in this study were approved by the Institutional Animal Care and Use Committee (IACUC) of Boston University Medical campus (approved protocol number: AN-15222), and all efforts were made to minimize suffering animals. This study was carried out in strict accordance with the recommendations in the Guide for the Care and Use of Laboratory Animals of the National Institutes of Health.

### Cell culture

Human embryonic kidney 293 cells (Clontech) and mouse embryonic fibroblasts (MEFs) were maintained in Dulbecco’s modified Eagle’s medium (DMEM; Life Technologies). Mouse MC3T3-E1 osteoblastic cells subclone 4 (ATCC CRL-2593) were maintained in α-minimum essential medium (α-MEM; Life Technologies). Media were all supplemented with 10% fetal bovine serum (FBS; Sigma-Aldrich), 100 units/ml penicillin and 100 μg/ml streptomycin in a 5% CO_2_ atmosphere at 37 °C. The media were refreshed twice a week.

### RNA extraction and quantitative real-time PCR

To investigate the gene expression of FAM20 family members in various tissues, total RNA was extracted with TRIzol reagent (Life Technologies) using mouse tissues, i.e. brain, heart, lung, kidney, calvaria, and tooth (C57BL/6, 5-week-old, male). Two μg of the total RNA extract was used for RT using the Omniscript RT Kit (Qiagen). Real-time PCR was performed in triplicate using the specific primers-probe for mouse *Fam20a* (Applied Biosystems, ABI assay number; Mm00464089_m1), mouse *Fam20b* (Mm01286165_m1), mouse *Fam20c* (Mm00504210_m1), or rodent *glyceraldehyde-3-phosphate dehydrogenase* (*Gapdh*; 04308313), and the expression levels were analyzed by the ABI 7300 real-time PCR system. The mean fold changes in the expression of each *Fam20* member relative to that of *Gapdh* were calculated using the values obtained from the brain cDNA with *Fam20a* expression as a calibrator by means of 2^−ΔΔ CT^ method as we previously reported^39^. Three independent experiments were performed and the results were essentially identical.

### Immunohistochemistry

To determine the localization of endogenous FAM20A protein *in vivo*, formalin fixed paraffin embedded sections of non-diseased mouse heads (C57BL/6, 4-week-old, male) were used to perform immunohistochemical staining as we reported^39^,^40^. Sections were incubated with anti-FAM20A (goat polyclonal, Santa Cruz Biotech, catalog number: sc-164308, diluted in 1:800), anti-FAM20C antibody (goat polyclonal, Santa Cruz Biotech, catalog number: sc-160322, diluted in 1:200), or non-immune goat serum as a negative control. The sections counterstained with hematoxylin were photographed under a light microscope.

### Generation of FAM20A and FAM20C forms in mammalian expression vectors

All vector construction was performed by polymerase chain reaction (PCR) using HotStarTaq DNA polymerase (Qiagen) and site-directed mutagenesis with the megaprimer PCR procedure^41^. The plasmids containing the full length sequence of human FAM20A and FAM20C were purchased from OriGene and used as PCR templates. The primer sequences used in this study were described in Table 1. The coding region of wild type (WT) FAM20A without stop codon was amplified by PCR (forward-reverse primers; F1-R1, Table 1) and the amplified fragment was subcloned into pcDNA3-3′-Flag-tag (pc3-Flag) and pcDNA3-3′-HA-tag (pc3-HA) mammalian expression vectors. The coding region of WT FAM20C was amplified by the primers (F5-R6), and it was subcloned into pc3-HA and pcDNA3.1-3′-V5/His-tag (pc3.1-V5/His) vectors. The plasmids harboring pc3-FAM20A WT-Flag, pc3-FAM20A WT-HA, pc3-FAM20C WT-HA and pc3.1-FAM20C WT-V5/His were then obtained. To generate AI form of FAM20A with c.1175_1179delGGCTC mutation^3^ in the expression vector, which resulted in the frame shift mutation at arginine residue 392 to proline followed by 21 extra amino acids and the premature stop codon (p.R392PfsX22), the forward and reverse primers with this mutation (F2 and R2) and the reverse primer corresponding to the extra amino acids without premature stop codon (R3) were designed, thus generating FAM20A R392Pfs21. The PCR fragment of FAM20A R392Pfs21 was amplified by the primers (F1-R3) using (F1-R2) and (F2-R3) fragments by means of megaprimer PCR method, and the resulted F1-R3 fragment was subcloned into pc3-Flag. To create another AI form of FAM20A with c.992G >A mutation^4^, which resulted in the missense mutation at glycine residue 331 to aspartic acid (p.G331D), the forward and reverse primers with this missense mutation were designed (F3 and R4). The PCR fragment of FAM20A G331D was amplified by the primers (F1-R1) using (F1-R4) and (F3-R1) fragments by means of megaprimer PCR method, and the resulted F1-R1 fragment was subcloned into pc3-Flag. The plasmids harboring pc3-FAM20A R392Pfs21-Flag and pc3-FAM20A G331D-Flag were then obtained. We also generated the kinase inactive form of FAM20C (FAM20C D478A) and one of the Raine syndrome-mutant form of FAM20C (FAM20C P328S) in the expression vectors (pc3.1-FAM20C D478A-V5 and pc3.1-FAM20C P328S-V5) according to the previous report^20^ (F6 and R7 for FAM20C D478A, and F7 and R8 for FAM20C P328S). All plasmids generated were sequenced, and their DNA sequences were 100% identical to the respective reference DNA sequences available at NCBI or the respective mutations.

### Binding of FAM20A to FAM20C and the effect of FAM20A on FAM20C localization

The 293 cells were plated onto 6 well culture plates at a density of 3 × 10^5^ cells/well and transfected with pc3-FAM20A WT-Flag and pc3-FAM20C-HA. After 24 hours, cells were lysed and then immunoprecipitated with anti-Flag antibody and Western blot (WB) analysis was performed with anti-HA antibody (clone 3F10, Roche Diagnostics) to investigate the interaction between FAM20A and FAM20C. The expression of FAM20C-HA or FAM20A-Flag was also verified using the same cell lysates by immunoprecipitation (IP)-WB analysis with anti-HA or anti-Flag antibody, respectively. To determine the interaction between FAM20A and mutant forms of FAM20C, 293 cells were transfected with pc3-FAM20A WT-HA and pc3.1-FAM20C WT-V5, pc3.1-FAM20C D478A-V5, or pc3.1-FAM20C P328S-V5. Cell lysates were used for immunoprecipitation with anti-V5 antibody and WB analysis was performed to detect the interaction with anti-HA antibody. The expression of FAM20A WT-HA, or FAM20C WT-V5 FAM20C D478A-V5, or FAM20C P328S-V5 was verified using the same lysates by WB analysis with anti-HA or IP-WB analysis with anti-V5 antibody. Chemiluminescent detection of bound antibodies was determined using the ECL Western blotting detection reagents (Amersham ECL Prime, GE Healthcare Life Sciences).

To investigate the effect of FAM20A co-transfection on the localization of FAM20C, 293 cells were plated in the same manner as described and transfected with pc3-FAM20A WT-Flag, pc3-FAM20A R392Pfs21-Flag, or pc3-FAM20A G331D-Flag in the presence of pc3-FAM20C-HA. The conditioned media and cell lysates were collected and analyzed by IP-WB analysis in the same manner as described above.

### Generation of recombinant human Glutathione-S-Transferase (GST)-FAM20A fusion protein

The primer sequences of FAM20A cDNA corresponding to its mature protein (residues 22-541; Asp^22^-Ser^541^) were described (Table 1, F4-R5). The PCR products were amplified, cloned into pGEX6P-1 vector (GE Healthcare Life Sciences), sequenced, and the vector that expresses the mature FAM20A protein (pGEX-FAM20A) was obtained. The pGEX-FAM20A or pGEX empty vector was transformed into the BL21-CodonPlus bacterial strain (Agilent Technologies), cultured, and the synthesis of GST-FAM20A fusion protein or GST protein alone was induced by isopropyl-D-1-thiogalactopyranoside (IPTG). The bacteria were further cultured overnight at 20 °C. The cultures were then centrifuged and lysed in PBS containing 1% Triton X-100. After the centrifugation, the supernatants were incubated with glutathione-sepharose beads (GE Healthcare Life Sciences) overnight and the beads were extensively washed with PBS. The beads were then treated with the elution buffer (10mM glutathione, 50mM Tris-HCl pH8.0) to release the conjugated proteins. The eluted proteins were pooled, dialyzed against distilled water, lyophilized and resuspended in distilled water. The protein concentration of each GST protein was measured using a Detergent Compatible (DC) protein assay kit (Bio-Rad) and a plate reader. Aliquots of each GST protein were mixed with SDS sample buffer, applied to 4–12% SDS-PAGE, and stained with Coomassie Brilliant Blue R-250 (CBB) (Bio-Rad). An aliquot of the GST-FAM20A protein generated was subjected to WB analysis with anti-FAM20A or anti-GST (goat polyclonal, GE Healthcare Life Sciences, product code: 27-4577-01) antibody and the immunoreactivity of the protein was confirmed.

### Generation of recombinant human FAM20C WT- and D478A-V5/His proteins

Recombinant human (rh) FAM20C WT- and D478A-V5/His proteins were generated and purified in the same manner as we previously reported^40^. Briefly, 293 cells were transfected with the pc3.1-FAM20C WT- or D478A-V5/His vector, and the clones derived from G418-resistant cells were isolated. Conditioned media were collected from each clone (out of 10 clones), and IP-WB analysis was performed with anti-V5 antibody. The clone which synthesized the highest level of rhFAM20C-V5/His protein was further cultured, the conditioned media were collected, and rhFAM20C WT- or D478A-V5/His protein was purified using a Ni-NTA agarose resin (Qiagen). The purified each rhFAM20C protein was pooled, dialyzed against distilled water, lyophilized and dissolved in distilled water. The protein concentration was measured using a DC protein assay kit and it was kept at −20 °C until use. The purity of the rhFAM20C WT- or D478A-V5/His protein was assessed by 4–12% SDS-PAGE stained with CBB and by WB analyses with anti-V5 and anti-FAM20C antibodies. The presence of hFAM20C was also confirmed by Mass Spectrometry analysis (data not shown).

### GST pull-down assay

To investigate the direct interaction between FAM20A and FAM20C, GST protein or GST-FAM20A protein was incubated with rhFAM20C WT- or D478A-V5/His overnight at 4 °C. After addition of glutathione-sepharose beads, the samples were washed with lysis buffer twice, mixed with SDS sample buffer, applied to 4–12% SDS-PAGE and WB analysis was performed with anti-V5 antibody. The expression of GST proteins (GST and GST-FAM20A) used for GST pull-down as input was analyzed by Western blotting with anti-GST antibody.

### Isolation and primary cell culture of mouse embryonic fibroblasts (MEFs)

Heterozygous *Fam20a* KO mice previously generated^35^ were intercrossed and the embryos at 14.5 days were dissected. Genomic DNA was extracted to determine the genotypes of embryos by PCR as previously established^35^ and the embryos were further minced. Cells were released by treating the minced samples with trypsin for 1 hour at 37 °C and plated onto the culture plates. Only 2–5 passages of MEF cells were used for the experiments.

### FAM20C expression in *Fam20a* KO MEF cells

MEF cells from *Fam20a* WT and KO embryos were plated and cultured for 48 hrs. Conditioned media were collected and the expression of extracellular FAM20C was then analyzed by IP-WB analysis with anti-FAM20C antibody. Cells were lysed and cell lysates were analyzed by Western blotting using anti-FAM20C and anti-FAM20A antibodies to verify the expression levels of intracellular FAM20C and FAM20A in the cell lysates. The expression level of β-TUBULIN as a loading control in the cell lysates was measured by WB analysis with anti-β-TUBULIN antibody (mouse monoclonal, BD Biosciences, catalog number: 556321). Films with non-saturated exposure were used and densitometric analysis was carried out using Image-J software (NIH). The mean gray value intensity was quantified by measuring the expression of intracellular FAM20C and β-TUBULIN, and the value intensity of intracellular FAM20C was normalized to that of β-TUBULIN in each cell type using the same cell lysate. Three independent experiments were performed and data are represented as the mean + standard deviation (SD) with statistical analysis.

### *In vitro* mineralization assay and Alizarin Res S staining quantification

To collect the conditioned media from *Fam20a* KO and WT MEFs, cells were plated onto 15 cm culture dishes. After cells reached confluence, the serum was starved for 24 hours and the conditioned media were collected. The media were centrifuged at 1,500 rpm for 5 min to remove any dead cells/cellular debris, and kept at 4 °C until use. MC3T3-E1 cells were plated onto 35 mm culture dishes in triplicates at a density of 3 × 10^5^ cells/dish. After cells reached confluence, the media were refreshed twice a week as follows; 50% v/v of the conditioned media from *Fam20a* WT or KO MEFs, and 50% v/v of α-MEM containing 20% of FBS and antibiotics described above in the presence of 50 μg/ml of ascorbic acid and 4 mM of β-glycerophosphate. To visualize the mineralized nodules *in vitro*, Alizarin Red S staining was performed as previously reported^42^. Alizarin Red S dye deposited onto the cell matrices was extracted with 10% cetyl pyridinium chloride buffer in 10 mM sodium phosphate. Absorbance was measured at 560 nm, Alizarin Red S concentration in each sample was calculated using an Alizarin Red S standard curve, and the values are expressed as the mean + SD (n = 3) with statistical analysis.

### Statistical analysis

Student T-test was used to assess the statistical significance between groups. P value with less than 0.01 was considered significant.

## Additional Information

**How to cite this article**: Ohyama, Y. *et al*. FAM20A binds to and regulates FAM20C localization. *Sci. Rep.* **6**, 27784; doi: 10.1038/srep27784 (2016).

## Supplementary Material

Supplementary Table 1

## Figures and Tables

**Table 1:** Primer sequences.

Primer name		Sequence
Human FAM20A WT	F1	5′-GCGGTACCATGCCGGGGCTGCGCCGGGAC-3′
Human FAM20A WT	R1	5′-GCCTCGAGGCTTGTCAAGTTAGCCTG-3′
Human FAM20A R392Pfs21	F2	5′-CAACAACAGCCAGCCTCAATGTCATCG-3′
Human FAM20A R392Pfs21	R2	5′-CGATGACATTGAGGCTGGCTGTTGTTG-3′
Human FAM20A R392Pfs21	R3	5′-GCCTCGAGTAATGGTGCCGGTCCATATTC-3′
Human FAM20A G331D	F3	5′-GTATGCTGTCTGTGACAACCCACACCTG-3′
Human FAM20A G331D	R4	5′-CAGGTGTGGGTTGTCACAGACAGCATAC-3′
GST-human FAM20A WT	F4	5′-GCCAATTGGACCTCTACTTCCACCTCTGG-3′
GST-human FAM20A WT	R5	5′-GCCTCGAGTTAGCTTGTCAAGTTAGCCTG-3′
Human FAM20C WT	F5	5′-GCGGTACCGCCATGAAGATGATGCTGG-3′
Human FAM20C WT	R6	5′-GCCTCGAGCCTCGCCGAGGCGGCTCTG-3′
Human FAM20C D478A	F6	5′-CACTTAGCCAATGGAAGAGGG-3′
Human FAM20C D478A	R7	5′-CCCTCTTCCATTGGCTAAGTG-3′
Human FAM20C P328S	F7	5′-CGGGTCCCTTCCGTGGCCGGC-3′
Human FAM20C P328S	R8	5′-GCCGGCCACGGAAGGGACCCG-3′

Primer sequences used in this study. F; Forward primer, R; Reverse primer.
