# Supplementary material for: FAM20A binds to and regulates FAM20C localization
Source: Sci Rep. 2016 Jun 13;6:27784. doi: 10.1038/srep27784 (PMC4904241; doi:10.1038/srep27784)
Supplement: Supplementary Table 1 [file srep27784-s1.pdf]

	Fam20a	Fam20b	Fam20c
**Brain**	1.10±0.10	1.23±0.01	3.85±0.15 *
**Heart**	1.23±0.12	4.86±0.66 *	4.04±0.37 *
**Lung**	7.88±0.78 **	20.64±2.51 **	20.59±1.78 **
**Kidney**	0.83±0.00	3.13±0.13 *	9.57±0.40 **
**Calvaria**	8.06±1.30 **	3.13±0.55	5.24±0.64 *
**Tooth**	10.08±0.46 **	2.34±0.12	9.36±0.42 **

S1 Table. Numerical data of expression of Fam20 family members in various tissues by real-time PCR. The numerical data of real-time PCR analysis in Figure 1A with statistical analysis were shown. The asterisk indicates the presence of statistical difference of the extent of expression level based on the expression of *Fam20a* in brain. *; P<0.05, **; p<0.01.
